# Ethyl *N*-[(benz­yloxy)thio­carbon­yl]carbamate

**DOI:** 10.1107/S1600536811055942

**Published:** 2012-01-07

**Authors:** Sung Kwon Kang, Nam Sook Cho, Min Kyeong Jeon

**Affiliations:** a Department of Chemistry, Chungnam National University, Daejeon 305-764, Republic of Korea

## Abstract

In the title compound, C_11_H_13_NO_3_S, the dihedral angle between the benzyl and carbamate groups is 12.67 (10)°. The S atom and the carbonyl O atom are positioned *anti* to each other. In the crystal, pairs of N—H⋯S hydrogen bonds link mol­ecules into inversion dimers.

## Related literature

For the synthesis and reactivity of pyrimidine and its derivatives, see: Cho *et al.* (1996 ▶); Ra *et al.* (1999 ▶). For the synthesis of oxadiazole derivatives, see: Renaut *et al.* (1991 ▶).
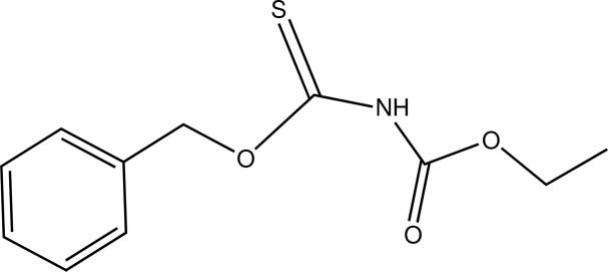


## Experimental

### 

#### Crystal data

C_11_H_13_NO_3_S
*M* *_r_* = 239.28
Triclinic, 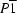

*a* = 6.608 (4) Å
*b* = 7.963 (7) Å
*c* = 12.369 (6) Å
α = 87.451 (13)°
β = 80.725 (17)°
γ = 77.324 (18)°
*V* = 626.7 (7) Å^3^
*Z* = 2
Mo *K*α radiation
μ = 0.25 mm^−1^
*T* = 296 K
0.26 × 0.24 × 0.15 mm

#### Data collection

Bruker SMART CCD area-detector diffractometer
Absorption correction: multi-scan (*SADABS*; Bruker, 2002 ▶) *T*_min_ = 0.927, *T*_max_ = 0.956
13629 measured reflections
2930 independent reflections
2143 reflections with *I* > 2σ(*I*)
*R*_int_ = 0.054

#### Refinement

*R*[*F* ^2^ > 2σ(*F* ^2^)] = 0.036
*wR*(*F* ^2^) = 0.106
*S* = 1.02
2930 reflections
149 parameters
H atoms treated by a mixture of independent and constrained refinement
Δρ_max_ = 0.21 e Å^−3^
Δρ_min_ = −0.20 e Å^−3^

### 

Data collection: *SMART* (Bruker, 2002 ▶); cell refinement: *SAINT* (Bruker, 2002 ▶); data reduction: *SAINT*; program(s) used to solve structure: *SHELXS97* (Sheldrick, 2008 ▶); program(s) used to refine structure: *SHELXL97* (Sheldrick, 2008 ▶); molecular graphics: *ORTEP-3* (Farrugia, 1997 ▶); software used to prepare material for publication: *WinGX* (Farrugia, 1999 ▶).

## Supplementary Material

Crystal structure: contains datablock(s) global, I. DOI: 10.1107/S1600536811055942/tk5038sup1.cif

Structure factors: contains datablock(s) I. DOI: 10.1107/S1600536811055942/tk5038Isup2.hkl

Supplementary material file. DOI: 10.1107/S1600536811055942/tk5038Isup3.cml

Additional supplementary materials:  crystallographic information; 3D view; checkCIF report

## Figures and Tables

**Table 1:** Hydrogen-bond geometry (Å, °)

*D*—H⋯*A*	*D*—H	H⋯*A*	*D*⋯*A*	*D*—H⋯*A*
N11—H11⋯S10^i^	0.83 (2)	2.66 (2)	3.481 (2)	174.3 (17)

Symmetry code: (i) 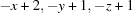.

**Fig. 1.**
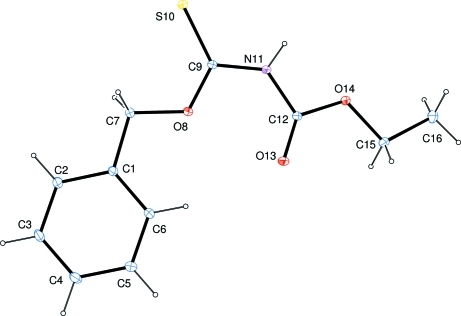
Molecular structure of (I), showing the atom-numbering scheme and 30% probability ellipsoids.

**Fig. 2.**
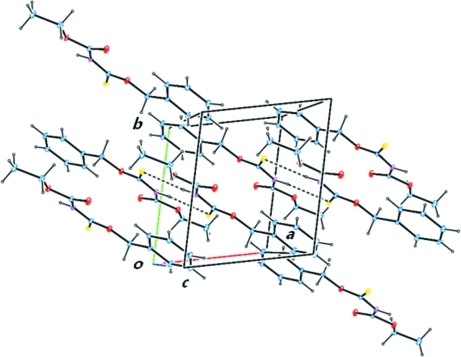
Part of the crystal structure of (I), showing molecules linked by intermolecular N—H···S hydrogen bonds (dashed lines).

## supplementary crystallographic information

### Comment

Within the framework of our systematic studies to obtain new analogues of pyrimidine and their derivatives, we reported the synthesis and reactivity of 5-amino-2*H*-1,2,4-thiadiazolin-3-one and 5-amino-2*H*-1,2,4-oxadiazolin-3-one (Cho *et al.*, 1996; Ra *et al.*, 1999). These compounds are 5-membered analogues of cytosine on the basis of the well known analogy between a –CH═ CH– group in a benzenoid hydrocarbon and either the divalent oxygen or sulfur in their oxygen- and sulfur-containing counterparts, respectively. On the same basis, 1,2,4-oxadiazolidine-3,5-dione is 5-membered analog of uracil. The title compound, ethyl *N*-(Benzyloxythiocarbonyl)carbamate (I) is an intermediate for the formation of 3-benzyloxy-1,2,4-oxadiazole-5-one and 1,2,4-oxadiazolidine-3,5-dione (Renaut *et al.*, 1991).

In (I), the benzyl unit is almost planar, with an r.m.s. deviation of 0.020 Å from the corresponding least-squares plane defined by the seven constituent atoms. The dihedral angle between the benzyl unit (C1—C7 atoms) and the carbamate group (N11—O14 atoms) is 12.67 (10)°. The S10 and carbonyl-O13 atoms are positioned *anti* to each other (Fig. 1). The intermolecular N11—H···S10^i^ [symmetry codes: (i) -*x* + 2, -*y* + 1, -*z* + 1] hydrogen bonds link two molecules into a centrosymmetric dimer (Fig. 2 and Table 1), which stabilizes the crystal structure.

### Experimental

Benzyl alcohol (BnOH, 11.8 ml, 114 mmol) was added under N_2_, drop-wise, at r.t., over 15 min to a solution of ethyl isothiocyanatoformate (13.54 g, 103 mmol) in EtOAc (200 ml). After refluxing for 5 h, the solvent was evaporated under reduced pressure. The crude yellow solid obtained was stirred with Et_2_O (25 ml) for 2 h and placed at -18 °C overnight. The precipitated product was isolated by suction and washed with hexane. Colourless crystals of (I) were obtained from its DMSO solution by slow evaporation of the solvent at room temperature.

### Refinement

Atom H11 of the NH group was located from a difference Fourier map and refined freely [refined distance; N—H = 0.83 (2) Å]. Other H atoms were positioned geometrically and refined using a riding model, with C—H = 0.93–0.97 Å, and with *U*_iso_(H) = 1.2*U*_eq_(carrier C) for aromatic and methylene, and 1.5*U*_eq_(carrier C) for methyl H atoms.

### Crystal data

**Table:**

C_11_H_13_NO_3_S	*Z* = 2
*M**_r_* = 239.28	*F*(000) = 252
Triclinic, *P*1	*D*_x_ = 1.268 Mg m^−^^3^
Hall symbol: -P 1	Mo *K*α radiation, λ = 0.71073 Å
*a* = 6.608 (4) Å	Cell parameters from 3416 reflections
*b* = 7.963 (7) Å	θ = 2.6–27.5°
*c* = 12.369 (6) Å	µ = 0.25 mm^−^^1^
α = 87.451 (13)°	*T* = 296 K
β = 80.725 (17)°	Block, colourless
γ = 77.324 (18)°	0.26 × 0.24 × 0.15 mm
*V* = 626.7 (7) Å^3^	

### Data collection

**Table:**

Bruker SMART CCD area-detector diffractometer	2143 reflections with *I* > 2σ(*I*)
graphite	*R*_int_ = 0.054
φ and ω scans	θ_max_ = 27.7°, θ_min_ = 1.7°
Absorption correction: multi-scan (*SADABS*; Bruker, 2002)	*h* = −8→8
*T*_min_ = 0.927, *T*_max_ = 0.956	*k* = −10→10
13629 measured reflections	*l* = −16→16
2930 independent reflections	

### Refinement

**Table:**

Refinement on *F*^2^	Primary atom site location: structure-invariant direct methods
Least-squares matrix: full	Secondary atom site location: difference Fourier map
*R*[*F*^2^ > 2σ(*F*^2^)] = 0.036	Hydrogen site location: inferred from neighbouring sites
*wR*(*F*^2^) = 0.106	H atoms treated by a mixture of independent and constrained refinement
*S* = 1.01	*w* = 1/[σ^2^(*F*_o_^2^) + (0.0609*P*)^2^] where *P* = (*F*_o_^2^ + 2*F*_c_^2^)/3
2930 reflections	(Δ/σ)_max_ = 0.001
149 parameters	Δρ_max_ = 0.21 e Å^−^^3^
0 restraints	Δρ_min_ = −0.19 e Å^−^^3^

### Special details

**Table:**

Geometry. All s.u.'s (except the s.u. in the dihedral angle between two l.s. planes) are estimated using the full covariance matrix. The cell s.u.'s are taken into account individually in the estimation of s.u.'s in distances, angles and torsion angles; correlations between s.u.'s in cell parameters are only used when they are defined by crystal symmetry. An approximate (isotropic) treatment of cell s.u.'s is used for estimating s.u.'s involving l.s. planes.
Refinement. Refinement of *F*^2^ against ALL reflections. The weighted *R*-factor *wR* and goodness of fit *S* are based on *F*^2^, conventional *R*-factors *R* are based on *F*, with *F* set to zero for negative *F*^2^. The threshold expression of *F*^2^ > 2σ(*F*^2^) is used only for calculating *R*-factors(gt) *etc*. and is not relevant to the choice of reflections for refinement. *R*-factors based on *F*^2^ are statistically about twice as large as those based on *F*, and *R*- factors based on ALL data will be even larger.

### Fractional atomic coordinates and isotropic or equivalent isotropic displacement parameters (Å^2^)

**Table:**

	*x*	*y*	*z*	*U*_iso_*/*U*_eq_	
C1	0.2095 (2)	0.85565 (18)	0.30806 (11)	0.0469 (3)	
C2	0.0200 (2)	0.9802 (2)	0.33462 (13)	0.0618 (4)	
H2	−0.0073	1.0385	0.4007	0.074*	
C3	−0.1272 (2)	1.0161 (2)	0.26156 (15)	0.0694 (5)	
H3	−0.2525	1.0962	0.2802	0.083*	
C4	−0.0860 (2)	0.9320 (2)	0.16136 (15)	0.0686 (5)	
H4	−0.184	0.9554	0.1135	0.082*	
C5	0.1039 (2)	0.8120 (2)	0.13283 (14)	0.0638 (4)	
H5	0.1332	0.7582	0.0651	0.077*	
C6	0.2500 (2)	0.7728 (2)	0.20631 (13)	0.0546 (4)	
H6	0.3741	0.6915	0.1875	0.065*	
C7	0.3573 (2)	0.8143 (2)	0.39377 (12)	0.0578 (4)	
H7A	0.3882	0.9193	0.4172	0.069*	
H7B	0.292	0.7591	0.4573	0.069*	
O8	0.55155 (14)	0.69857 (13)	0.34312 (7)	0.0515 (3)	
C9	0.6988 (2)	0.63279 (18)	0.40536 (11)	0.0444 (3)	
S10	0.68670 (6)	0.67360 (6)	0.53914 (3)	0.05991 (16)	
N11	0.87013 (18)	0.52354 (16)	0.34747 (9)	0.0497 (3)	
H11	0.976 (3)	0.484 (2)	0.3762 (15)	0.070 (5)*	
C12	0.9048 (2)	0.47201 (19)	0.23650 (11)	0.0467 (3)	
O13	0.77902 (16)	0.49923 (16)	0.17264 (8)	0.0646 (3)	
O14	1.10760 (14)	0.38449 (13)	0.21449 (7)	0.0534 (3)	
C15	1.1791 (2)	0.3185 (2)	0.10216 (12)	0.0613 (4)	
H15A	1.1634	0.4125	0.0494	0.074*	
H15B	1.097	0.2379	0.0865	0.074*	
C16	1.4076 (3)	0.2296 (3)	0.09575 (16)	0.0833 (6)	
H16A	1.4598	0.1837	0.0237	0.125*	
H16B	1.4209	0.1377	0.1488	0.125*	
H16C	1.4872	0.3109	0.1107	0.125*	

### Atomic displacement parameters (Å^2^)

**Table:**

	*U*^11^	*U*^22^	*U*^33^	*U*^12^	*U*^13^	*U*^23^
C1	0.0442 (7)	0.0508 (8)	0.0428 (7)	−0.0034 (6)	−0.0082 (6)	0.0039 (6)
C2	0.0559 (9)	0.0692 (10)	0.0497 (8)	0.0089 (7)	−0.0075 (7)	0.0005 (7)
C3	0.0467 (8)	0.0775 (11)	0.0721 (11)	0.0127 (8)	−0.0126 (8)	0.0073 (9)
C4	0.0535 (8)	0.0833 (12)	0.0692 (11)	−0.0047 (8)	−0.0263 (8)	0.0083 (9)
C5	0.0608 (9)	0.0748 (11)	0.0571 (9)	−0.0073 (8)	−0.0218 (7)	−0.0050 (8)
C6	0.0459 (7)	0.0597 (9)	0.0561 (9)	−0.0007 (6)	−0.0152 (6)	−0.0059 (7)
C7	0.0519 (8)	0.0648 (9)	0.0479 (8)	0.0117 (7)	−0.0128 (6)	−0.0089 (7)
O8	0.0445 (5)	0.0661 (6)	0.0383 (5)	0.0052 (4)	−0.0127 (4)	−0.0044 (4)
C9	0.0422 (7)	0.0515 (8)	0.0388 (7)	−0.0043 (6)	−0.0118 (5)	−0.0008 (6)
S10	0.0581 (2)	0.0760 (3)	0.0394 (2)	0.00832 (19)	−0.01693 (16)	−0.01291 (18)
N11	0.0424 (6)	0.0670 (8)	0.0363 (6)	0.0024 (6)	−0.0146 (5)	−0.0053 (5)
C12	0.0438 (7)	0.0568 (8)	0.0384 (7)	−0.0040 (6)	−0.0112 (5)	−0.0031 (6)
O13	0.0510 (6)	0.0927 (8)	0.0468 (6)	0.0035 (5)	−0.0210 (5)	−0.0135 (6)
O14	0.0451 (5)	0.0698 (7)	0.0399 (5)	0.0050 (5)	−0.0115 (4)	−0.0113 (5)
C15	0.0581 (8)	0.0785 (11)	0.0414 (8)	0.0012 (8)	−0.0092 (7)	−0.0127 (7)
C16	0.0579 (9)	0.1058 (15)	0.0729 (12)	0.0090 (10)	−0.0010 (9)	−0.0257 (11)

### Geometric parameters (Å, °)

**Table:**

C1—C6	1.407 (2)	O8—C9	1.3402 (16)
C1—C2	1.418 (2)	C9—N11	1.3830 (18)
C1—C7	1.532 (2)	C9—S10	1.6864 (16)
C2—C3	1.408 (2)	N11—C12	1.4186 (18)
C2—H2	0.93	N11—H11	0.83 (2)
C3—C4	1.394 (2)	C12—O13	1.2160 (17)
C3—H3	0.93	C12—O14	1.3586 (17)
C4—C5	1.405 (2)	O14—C15	1.4745 (17)
C4—H4	0.93	C15—C16	1.512 (2)
C5—C6	1.407 (2)	C15—H15A	0.97
C5—H5	0.93	C15—H15B	0.97
C6—H6	0.93	C16—H16A	0.96
C7—O8	1.4708 (17)	C16—H16B	0.96
C7—H7A	0.97	C16—H16C	0.96
C7—H7B	0.97		
			
C6—C1—C2	118.77 (13)	C9—O8—C7	118.80 (11)
C6—C1—C7	123.26 (12)	O8—C9—N11	112.36 (12)
C2—C1—C7	117.95 (13)	O8—C9—S10	125.94 (10)
C3—C2—C1	120.36 (15)	N11—C9—S10	121.70 (10)
C3—C2—H2	119.8	C9—N11—C12	129.29 (11)
C1—C2—H2	119.8	C9—N11—H11	120.3 (13)
C4—C3—C2	120.30 (14)	C12—N11—H11	110.2 (13)
C4—C3—H3	119.9	O13—C12—O14	126.01 (13)
C2—C3—H3	119.9	O13—C12—N11	127.36 (12)
C3—C4—C5	119.82 (15)	O14—C12—N11	106.63 (11)
C3—C4—H4	120.1	C12—O14—C15	116.03 (10)
C5—C4—H4	120.1	O14—C15—C16	106.93 (13)
C4—C5—C6	120.23 (16)	O14—C15—H15A	110.3
C4—C5—H5	119.9	C16—C15—H15A	110.3
C6—C5—H5	119.9	O14—C15—H15B	110.3
C1—C6—C5	120.47 (13)	C16—C15—H15B	110.3
C1—C6—H6	119.8	H15A—C15—H15B	108.6
C5—C6—H6	119.8	C15—C16—H16A	109.5
O8—C7—C1	107.80 (12)	C15—C16—H16B	109.5
O8—C7—H7A	110.1	H16A—C16—H16B	109.5
C1—C7—H7A	110.1	C15—C16—H16C	109.5
O8—C7—H7B	110.1	H16A—C16—H16C	109.5
C1—C7—H7B	110.1	H16B—C16—H16C	109.5
H7A—C7—H7B	108.5		

### Hydrogen-bond geometry (Å, °)

**Table:**

*D*—H···*A*	*D*—H	H···*A*	*D*···*A*	*D*—H···*A*
N11—H11···S10^i^	0.83 (2)	2.66 (2)	3.481 (2)	174.3 (17)

Symmetry codes: (i) −*x*+2, −*y*+1, −*z*+1.
